# Direct *In Situ* Measurement of Quantum Efficiencies of Charge Separation and Proton Reduction at TiO_2_-Protected GaP Photocathodes

**DOI:** 10.1021/jacs.2c10578

**Published:** 2023-01-30

**Authors:** Zihao Xu, Bingya Hou, Fengyi Zhao, Sa Suo, Yawei Liu, Haotian Shi, Zhi Cai, Craig L. Hill, Djamaladdin G. Musaev, Matthew Mecklenburg, Stephen B. Cronin, Tianquan Lian

**Affiliations:** † Department of Chemistry, Emory University, 1515 Dickey Dr, Atlanta, Georgia30322, United States; ‡ ZJU-Hangzhou Global Scientific and Technological Innovation Center, Zhejiang University, Hangzhou, Zhejiang310014, China; § Department of Electrical Engineering, University of South California, 3710 McClintock Ave, Los Angeles, California90089, United States; ∥ Department of Chemistry, University of South California, 3710 McClintock Ave, Los Angeles, California90089, United States; ⊥ Cherry L. Emerson Centre for Scientific Computation, Emory University, 1515 Dickey Drive, Atlanta, Georgia30322, United States; # Core Center of Excellence in Nano Imaging (CNI), University of South California, 814 Bloom Walk, Los Angeles, California90089, United States

## Abstract

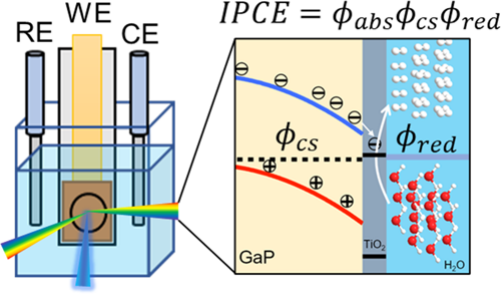

Photoelectrochemical solar fuel generation at the semiconductor/liquid interface consists of multiple elementary steps, including charge separation, recombination, and catalytic reactions. While the overall incident light-to-current conversion efficiency (IPCE) can be readily measured, identifying the microscopic efficiency loss processes remains difficult. Here, we report simultaneous *in situ* transient photocurrent and transient reflectance spectroscopy (TRS) measurements of titanium dioxide-protected gallium phosphide photocathodes for water reduction in photoelectrochemical cells. Transient reflectance spectroscopy enables the direct probe of the separated charge carriers responsible for water reduction to follow their kinetics. Comparison with transient photocurrent measurement allows the direct probe of the initial charge separation quantum efficiency (ϕ_CS_) and provides support for a transient photocurrent model that divides IPCE into the product of quantum efficiencies of light absorption (ϕ_abs_), charge separation (ϕ_CS_), and photoreduction (ϕ_red_), *i.e*., IPCE = ϕ_abs_ϕ_CS_ϕ_red_. Our study shows that there are two general key loss pathways: recombination within the bulk GaP that reduces ϕ_CS_ and interfacial recombination at the junction that decreases ϕ_red_. Although both loss pathways can be reduced at a more negative applied bias, for GaP/TiO_2_, the initial charge separation loss is the key efficiency limiting factor. Our combined transient reflectance and photocurrent study provides a time-resolved view of microscopic steps involved in the overall light-to-current conversion process and provides detailed insights into the main loss pathways of the photoelectrochemical system.

## Introduction

**Scheme 1:**
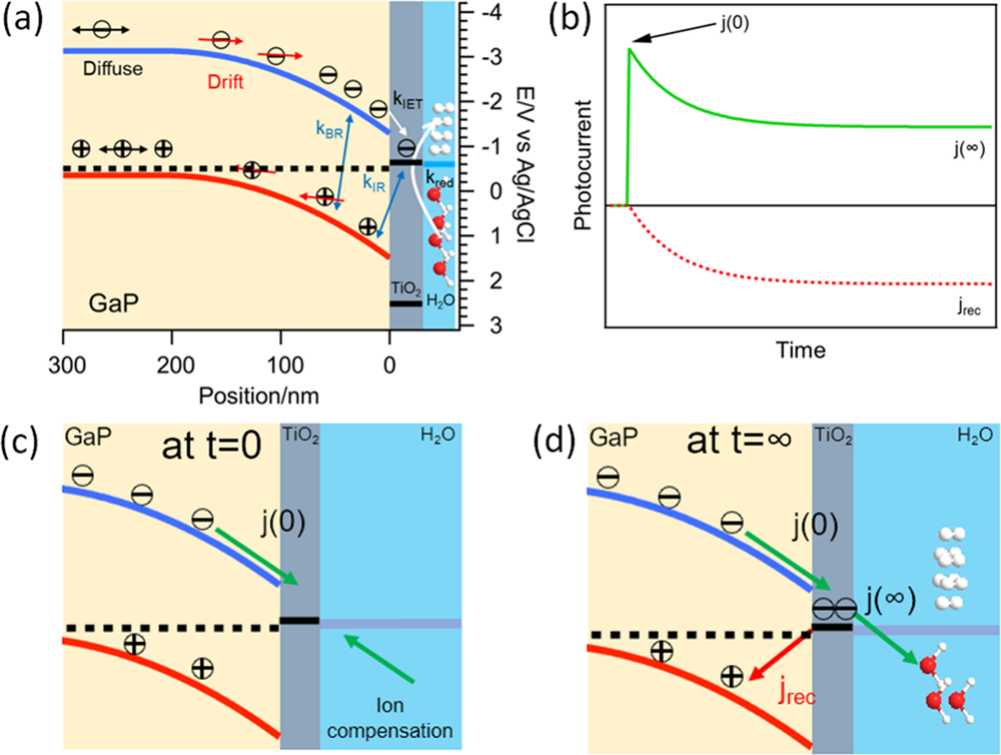
(a) Schematic Diagram of the GaP/TiO_2_ Junction under −0.5 V vs Ag/AgCl upon Photoexcitation, Showing a GaP Depletion Layer (Yellow), TiO_2_ ALD Layer (Gray, 5 nm), and Electrolyte (Blue). Both the GaP Conduction and Valence Bands Are Bent downwards by Applying a Bias. The Band Bending Drives the Photogenerated Electrons (Holes) towards (Away from) the GaP/TiO_2_ Interface. *k*_BR_: Bulk Recombination, *k*_IET_: Interfacial Electron Transfer from GaP to TiO_2_, *k*_IR_: Recombination across the GaP/TiO_2_ Interface, *k*_red_: Proton Reduction at the TiO_2_/Electrolyte Interface. (b) Cartoon of Transient Photocurrent in Response to Turning on of a CW Illumination Light, Showing the Peak Photocurrent, *j*(0), and Steady-State Photocurrent, *j*(∞). Also Shown in a Dashed Line Is the Growth of Interfacial Recombination Current That Is Responsible for the Decay of the Net Photocurrent. A Simplified Model for Describing the Transient Photocurrent at (c) the Initial Peak Current, j(0), and (d) Steady-State Current, *j*(∞). The Flow of *j*(0) Is Accompanied by the Ion Flow in Solution. The Accumulation of Surface Minority Carriers Leads to the Increase in Interfacial Recombination Loss and the Decrease in Photocurrent from the Initial to the Steady-State Value

Photoelectrochemical (PEC) systems capture sunlight and store its energy in the form of chemical bonds in fuels for on-demand electricity generation, mitigating the problems of sunlight intermittency.^1,2^ Semiconductors are often used as photon absorbers for oxygen and hydrogen evolution reactions (OER and HER) due to their suitable band gaps and superior light absorption and charge transport properties,^3−8^ and many recent studies show that their photoelectrode performance and photostability can be further enhanced by thin oxide, especially titanium oxide (TiO_2_) protection layers.^9−22^ In HER systems, the protective TiO_2_ layer is typically an *n*-type semiconductor due to oxygen vacancies, and it forms *p-n* junctions with *p-*type light-absorbing semiconductors that enhance charge separation.^16^ In these electrodes, such as gallium phosphate (GaP) electrodes protected by TiO_2_, the overall reaction consists of multiple elementary steps, including diffusion, drift, charge recombination within the GaP and at the GaP surface, charge separation and recombination across the GaP/TiO_2_ interface, and finally, catalytic reactions at the electrode/electrolyte interface driven by the separated charges, as illustrated in Scheme 1a.^9^ While the overall efficiency of light-to-current conversion can be characterized by current density and voltage (*J*–*V*) curves,^23,24^ it remains difficult to investigate how each microscopic elementary process contributes to the overall loss of the reaction and what the key efficiency limiting factors are.

Previously, a transient photocurrent method has been developed to understand the *J*–*V* characteristic of photoelectrodes.^23−27^ In this method, time-dependent photocurrent in response to the on/off modulation of light illumination is recorded, as shown in Scheme 1b. This response is often understood by a simplified model for the overall photoelectrochemical process. In this model, the reaction occurs on a surface site at the semiconductor/liquid interface; the peak current density j(0) is proportional to the efficiency of extracting photogenerated minority charge carriers from the bulk semiconductor to the interface (Scheme 1c); because of slow catalytic reaction rates, the photogenerated minority carriers are accumulated on the surface, increasing their interfacial recombination with majority carriers in the semiconductor (*j*_rec_) and decreasing the net photocurrent density until it reaches the steady-state value of *j*(*∞*), as depicted in Scheme 1d. Thus, the incident photon-to-current conversion efficiency (IPCE) under steady-state conditions can be defined by eq 1:
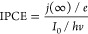
1where *e* is the elementary charge, *hv* is the photon energy, and *I*_0_ is the intensity of incident light intensity.

The transient photocurrent model divides the complex multistep overall light-to-current conversion process into three major sequential stages, and the overall IPCE and the quantum efficiencies of these stages can be expressed in eqs 2–4:^25−27^
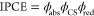
2
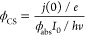
3
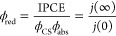
4ϕ_abs_ is the quantum efficiency of the incident photons absorption by the semiconductor; ϕ_CS_ is the charge separation quantum efficiency of transferring the photogenerated minority carriers to the surface to form long-lived carriers, which, as shown in Scheme 1a, reflects the competition of net interfacial electron transfer (*k*_IET_) with bulk and surface recombination processes within the semiconductor (*k*_BR_); ϕ_red_ is the photoreduction quantum efficiency of reducing surface species with the transferred minority carriers, which is determined by the competition of water reduction (*k*_red_) and interfacial charge recombination (*k*_IR_).

Although the transient photocurrent model has been used to rationalize the *J*–*V* characteristics of many photoelectrodes, testing the validity of such a model would require a nonphotocurrent-based probe that can measure the initial charge separation and/or photoreduction efficiencies. Thus, the development of *operando* time-resolved spectroscopic techniques that can directly probe these steps is essential to identify key efficiency limiting factors and to discover strategies for designing more efficient PEC systems. Recently, transient reflectance spectroscopy (TRS) has been applied to the study of carrier dynamics of photoelectrodes, including GaInP_2_, GaAs, Si, and GaP, with surface modification ranging from metal oxide protective layers to molecular species.^16,21,22,28^ In our previous work, we have successfully characterized GaP electrodes protected with TiO_2_ layers of varying thickness (GaP/TiO_2_) in air without external bias by TRS and showed that TRS can directly probe the kinetics of photogenerated carriers at the GaP/TiO_2_ junction.^28^ However, so far, these studies were carried out mainly *ex situ*, in the absence of applied bias and electrolyte, which cannot be directly connected to the performance of these materials for light-driven solar fuel generation reactions in photoelectrochemical cells.

In this work, we report a simultaneous *in situ* transient reflectance spectroscopy and photocurrent measurement of a photoelectrochemical cell with a 5 nm TiO_2_-protected GaP photocathode (GaP/TiO_2_, Scheme 1a). *In situ* transient reflectance spectroscopy enables the direct probe of the charge separation kinetics of photogenerated carriers. By comparing TRS and photocurrent, we can directly measure the charge separation efficiency and provide support for the transient photocurrent model that decomposes IPCE into the product of three sequential stages (IPCE = ϕ_abs_ϕ_CS_ϕ_red_). We investigate how the charge separation efficiency (ϕ_CS_) and photoreduction efficiency (ϕ_red_) depend on the applied bias and excitation fluence, and we identify key loss pathways of the overall light-driven HER on GaP/TiO_2_ photocathodes.

## Results

### Transient Photocurrent under CW Illumination

**Figure 1:**
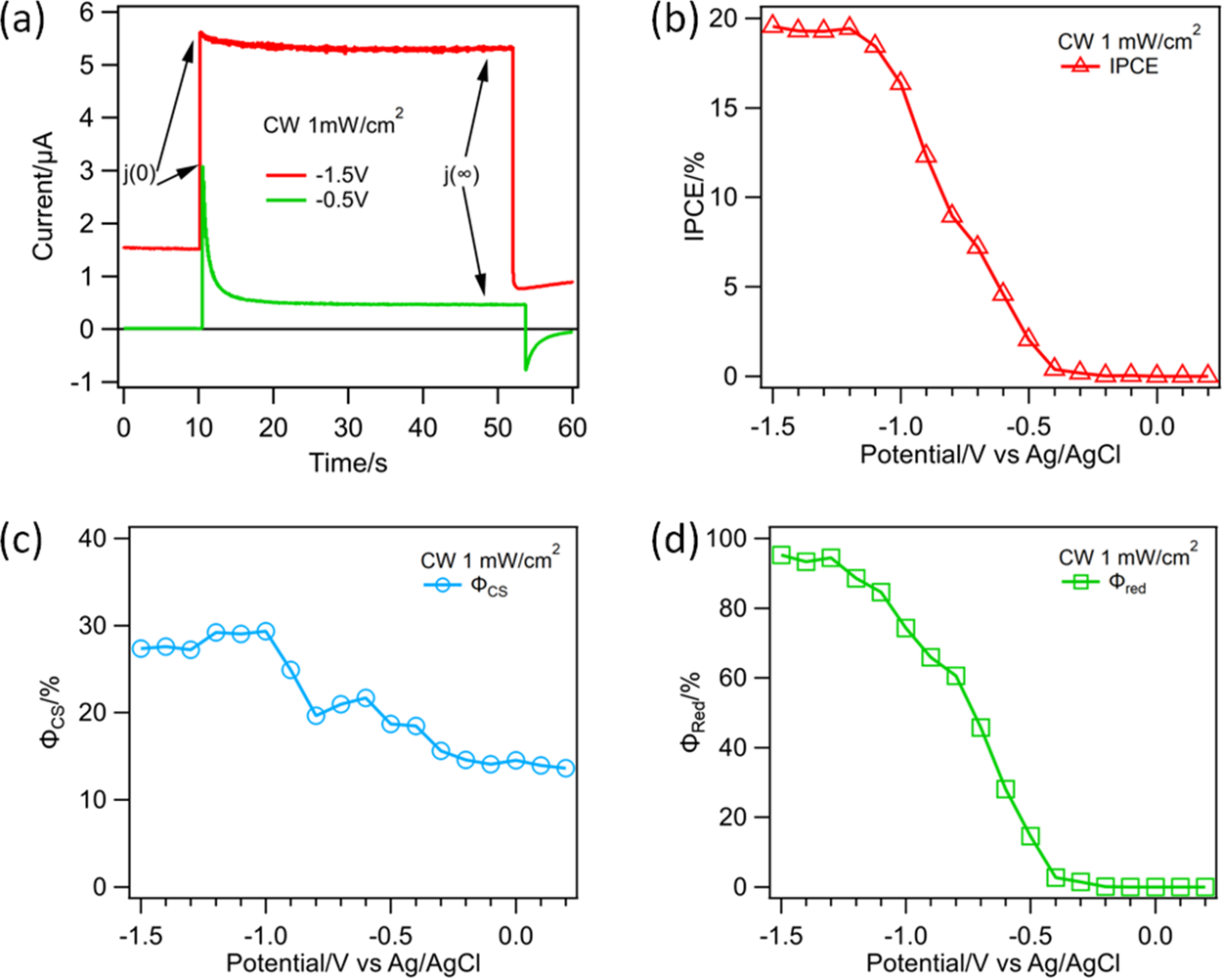
Transient photocurrent measurement under 1 mW/cm^2^ 405 nm CW LED illumination. (a) Current–time (*i*–*t*) curve under −1.5 and −0.5 V applied biases. The CW illumination is switched on at ∼10 s and off at ∼50 s. The dark current before illumination is subtracted from *j*(0) and *j*(*∞*) in the following calculation. (b) Measured IPCE as a function of applied potential. (c) The charge separation efficiency ϕ_CS_ and (d) photoreduction efficiency ϕ_red_ are calculated from the measured *j*(0) and *j*(*∞*).

We first carried out transient photocurrent study of GaP/TiO_2_ electrodes as a function of applied potential under modulated CW illumination. The details of photoelectrode preparation, TEM characterization (Figures S1 and S2), photocurrent measurement, and Mott–Schottky characterization (Figure S3) can be found in SI S1–S3. For GaP/TiO_2_ electrodes under these conditions, the photocurrent can be attributed to proton reduction.^14,29,30^ Shown in Figure 1a are the temporal evolution of photocurrent in response to the on and off modulation of continuous LED illumination at 405 nm at −1.5 V and −0.5 V (vs Ag/AgCl). Upon turning on the illumination, the photocurrent reaches a peak value *j*(0) and then decays to a steady-state value *j*(*∞*) on a ∼10 s time scale. IPCE values can be calculated from *j*(∞) according to eq 1. A representative IPCE measured under 1 mW/cm^2^ illumination power density and as a function of applied bias is shown in Figure 1b. Similar IPCE curves at lower CW illumination power densities of 0.04–0.12 mW/cm^2^ are shown in Figure S4. Within the range of CW illumination power densities, the IPCE values show negligible dependence on power density.

For GaP/TiO_2_ electrodes ϕ_abs_ is measured to be 75% under our experimental conditions for both 400 and 405 nm illumination light (see Figure S5). Following eqs 3 and 4, the values of ϕ_CS_ and ϕ_red_ as a function of applied bias measured at an illumination power density of 1 mW/cm^2^ are shown in Figure 1c,d, respectively. As shown in Figure 1b, the IPCE of the GaP/TiO_2_ electrode increases from ∼0 at a more positive applied potential of −0.4 V (Ag/AgCl) to a plateau value of ∼20% at a more negative potential of −1.2 V (Ag/AgCl). ϕ_red_ decreases from ∼100% at −1.5 V to ∼0% at −0.3 V, while ϕ_CS_ shows less pronounced bias dependence, decreasing from ∼30% at −1.5 V to ∼15% at 0 V. Comparison of Figure 1b, Figure 1c, and Figure 1d shows that the bias dependence of IPCE follows the trend of ϕ_red_.

### Transient Photocurrent under Pulsed Laser Illumination

**Figure 2:**
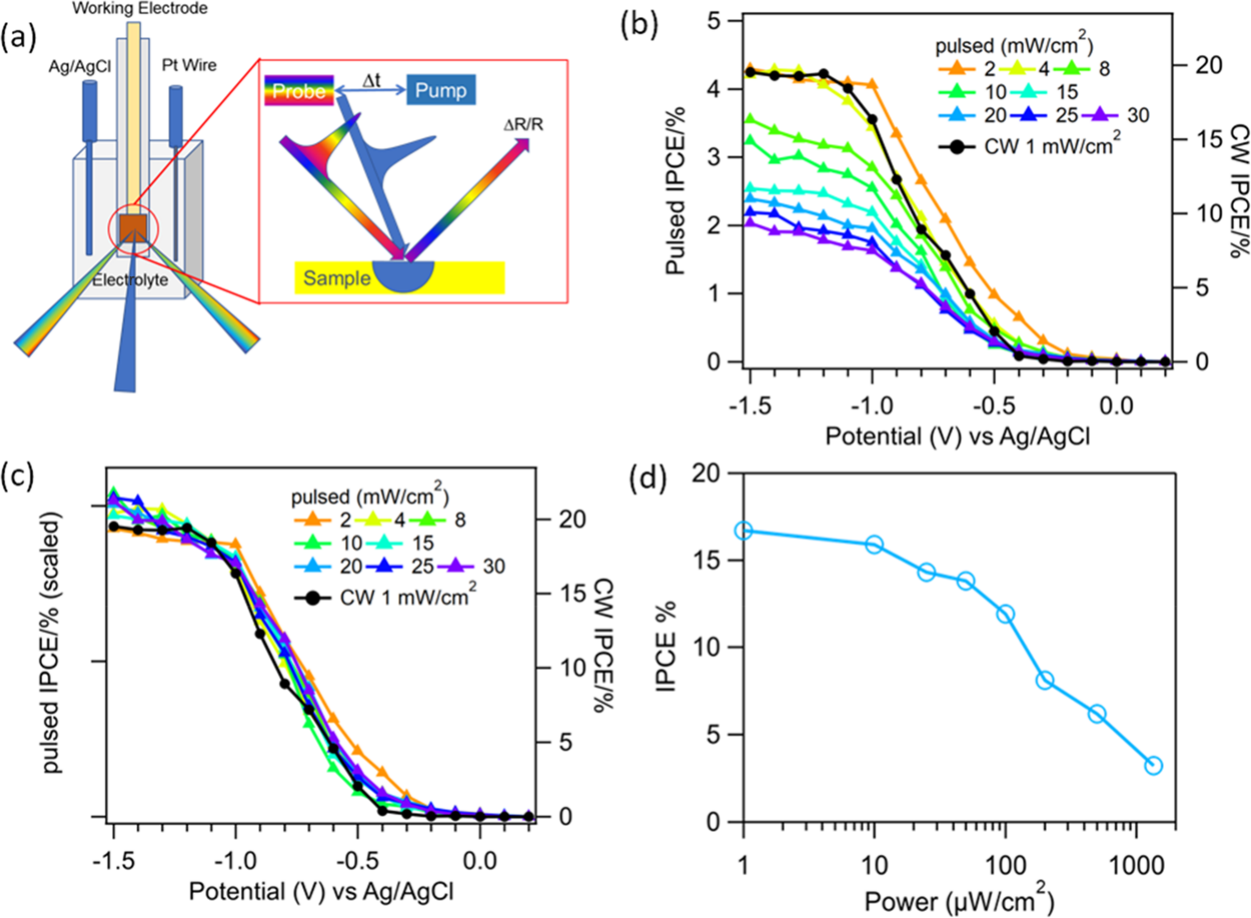
Simultaneous *in situ* transient reflectance and IPCE measurements with pulsed excitation at 400 nm. (a) Scheme of simultaneous photocurrent and *in situ* transient reflectance measurements of photogenerated carriers in GaP/TiO_2_ electrodes in a three-electrode photoelectrochemical cell. (b) Comparison and (c) normalized comparison of IPCE as a function of applied potential measured at indicated pulsed illumination power densities. Also shown in the comparison is the IPCE at CW illumination, plotted against the right axis. (d) IPCE as a function of average pulsed illumination power density at −1.5 V applied bias, showing that IPCE increases at lower average power density, approaching the value measured with CW illumination.

To provide experimental support for the three-stage transient current model described above, we carried out simultaneous *in situ* transient reflectance spectroscopy and transient photocurrent measurements of GaP/TiO_2_ photocathodes, as shown in Figure 2a. With this method, we will directly measure ϕ_CS_ by transient reflectance spectroscopy and compare with those determined from the transient photocurrent method. We first discuss the photocurrent results and will discuss the TRS results in the next section. Under the TRS conditions, the photocurrent is generated mainly by the pump beam at 400 nm because its pulse energy is much larger than the white light probe. A typical photocurrent transient is shown in Figure S6a. The measured IPCE values as a function of applied bias at indicated average illumination power densities (400 nm) are displayed in Figure 2b, and the normalized comparison of these curves are shown in Figure 2c. The normalized IPCE curves at different power densities agree well with each other, indicating that the illumination power affects the absolute IPCE value but not its dependence on the applied bias. Furthermore, these curves also resemble the IPCE curve measured under CW illumination (Figure 2c). The IPCE value at −1.5 V reduce from 4.6 to 2% as the average excitation power increases from 1 to 30 mW/cm^2^, significantly smaller than that of CW illumination (Figure 1b). Interestingly, as shown in Figure 2d, the IPCE increases further at a lower excitation power density and reaches the value of CW illumination at ∼1 μW/cm^2^ average power density. Unfortunately, to achieve sufficient signal-to-noise ratios, transient reflectance measurements (to be discussed below) were carried out at an average pulse excitation power density of 1 mW/cm^2^ and higher. These results show that compared to CW illumination, pulsed laser excitation reduces the absolute value of IPCE but does not affect its dependence on the applied bias. As will be discussed below, the key difference between the CW and pulsed illumination can be attributed to increased charge recombination loss caused by large transient photocarrier density within the ∼100 fs pulse width of the intense femtosecond illumination.

### *In Situ* Transient Reflectance Measurement of Charge Separation

**Figure 3:**
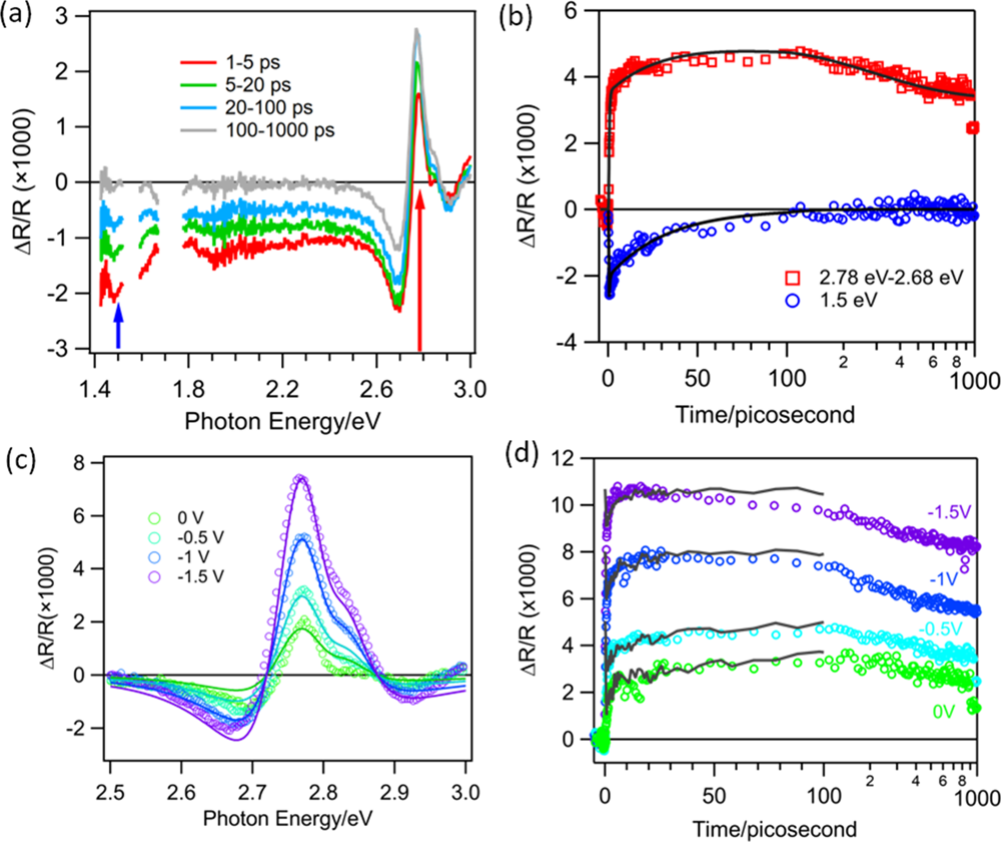
*In situ* transient reflectance measurements of charge separation spectra and kinetics with pulsed 400 nm excitation at an averaged power density of 15 mW/cm^2^. (a) Transient reflectance spectra at selected delay times under −0.5 V. Data around 1.55 and 1.7 eV have been removed due to probe intensity saturation. (b) Comparison of the kinetics of the free carrier signal at 1.5 eV (blue circles) and the Franz–Keldysh Oscillation (FKO) signal at 2.78 eV (red squares) and their multiexponential fits (solid lines). These kinetics are probed at the indicated positions of the blue and red arrows in panel (a). The FKO signal amplitude is obtained by the difference of the transient reflectance signal at 2.78 and 2.68 eV to remove the contribution of the free carrier signal in this region, and the following FKO amplitude and kinetics data are obtained by this method. (c) Averaged transient reflectance spectra in the FKO signal region at 100–200 ps delay time under indicated applied biases. The solid lines in (c) are fits of the FKO spectra to a model described in the main text and the SI. (d) Comparison of the kinetics of the FKO signal monitored at 2.78 eV subtracting 2.68 eV (circles) and the scaled free carrier signal (solid lines) at indicated applied biases. The free carrier signal has been displaced vertically and scaled such that the decay can be compared with the growth of the FKO signal.

Figure 3a shows the TR spectra of the GaP/TiO_2_ electrode under −0.5 V applied bias as a function of probe wavelength and pump–probe delay time after 400 nm excitation. For a delay time up to 100 ps, the TR spectra show two main features: (1) the decay of a broad negative Δ*R*/*R* signal from 1.4 to 3 eV and (2) the growth of an oscillatory signal centered at around 2.78 eV, the direct band gap of GaP.^31^ After 100 ps, only the oscillatory signal remains. The broad feature can be attributed to the reflectance decrease caused by the increase in free carrier density (Δ*N*) in the GaP, and the normalized reflectance change is given by , according to the Drude model.^32,33^ The oscillatory signal is assigned to the suppressed Franz–Keldysh Oscillation (FKO) signal, which is caused by the photoinduced change of the built-in electric field at the surface.^34−37^ Similar FKO signals have been reported recently on related TiO_2_-protected photoelectrodes.^16,21,28^

The probe penetration depth on the GaP ranges from 8 nm at 3.1 eV and 10 nm at 2.78 V to 21 nm at 1.5 eV (determined from the refractive index, see SI S6.1), making TR spectroscopy an interface sensitive approach. The FKO signal amplitude is directly proportional to the surface density of transferred electrons across the GaP/TiO_2_ junction (see further analysis below), and the free carrier signal amplitude is proportional to free carrier density within the GaP. These signals provide a direct probe of the kinetics of the initial transfer of free electrons in the GaP across the GaP/TiO_2_ interface. The transient reflectance kinetics of the free carrier decay (monitored at 1.5 eV) and FKO formation (monitored at 2.78 eV) are compared in Figure 3b. Both kinetics can be fit with a biexponential function with the same time constants. The fitting results are shown in Table S2. The time constants (and amplitudes) are 0.40 ± 0.02 ps (37.2%) and 32.6 ± 2.3 ps (62.8%) for the free carrier decay and 0.40 ± 0.02 ps (65.0%) and 32.6 ± 2.3 ps (35.0%) for the FKO growth. Similar agreement between the kinetics of free carrier decay and FKO signal formation is observed at all measured biases (see below and Table S2). These results confirm that the FKO signal growth can be attributed to the electron transfer across the GaP/TiO_2_*p-n* junction. It should be noted that we have used bi-exponential fits to obtain apparent time constants and confirm the agreement between the FKO and free carrier kinetics, while the actual kinetics of carrier transport and interfacial transfer is a much more complex process that requires numerical solution of transport equations to fit.^28^

Transient reflectance spectra and kinetics were measured as a function of applied potentials with an average excitation fluence of 15 mW/cm^2^ to investigate the bias dependence on the charge separation and recombination kinetics. A comparison of the average TR spectra at 100–200 ps (Figure 3c) shows that the FKO signal amplitude increases at more negative potentials from 0 to −1.5 V. The fitting of the FKO spectra is described in detail in SI S6.2 and eqs S6–S14, following our previous study on similar GaP/TiO_2_ junctions in the absence of externally applied bias,^28^ and the fitting parameters are listed in Table S1. This model assumes a weak field limit, in which the field modulation strength is much smaller than the damping rate of the transition, as shown in Table S1, and the FKO oscillation in the higher energy region is heavily damped, giving rise to “suppressed FKO” response.^35,38^ In the weak field limit, the FKO spectra shape is described by the third derivative of the primary spectra before the field perturbation, independent of the AC and DC field strengths (*E*_AC_ and *E*_DC_, respectively), and the amplitude of the FKO signal is given by Δ*R*/*R* (*ℏ*ω) ∝ *E*_DC_*E*_AC_.^28,34−37,39^ Because of the short penetration depth of the probe beam at 2.78 eV (∼10 nm), we have used the surface electric field amplitude as an approximation of the averaged field strength in the probe region for the FKO signal. With this approximation, *E*_DC_ is the built-in electric field in the depletion region in the dark at the GaP surface and *E*_AC_ is the light-induced change of the built-in field at the GaP surface. *E*_DC_ can be calculated by eq S5 from the measured flat band potential and dopant density (SI S3). *E*_AC_ at the GaP surface is proportional to the amount of separated photogenerated charge carriers across the GaP/TiO_2_*p-n* junction per unit electrode area (*E*_AC_ ∝ σ), following the Gauss law.^28^ This suggests that the FKO signal amplitude depends linearly on both the DC electrical field strength (or band bending) and the amount of separated carriers. This model provides reasonable fits to the FKO spectra at all applied biases, as shown in Figure 3c. Similar linear dependence of the Δ*R*/*R* (*ℏω*) signal on carrier density was also observed in a previous study.^21^ It is worth noting that this signal differs from strong field conditions, in which the FKO amplitude was reported to depend on the carrier density logarithmically.^16,22^ Furthermore, the presence of free carriers at early delay times can lead to band state filling and bandgap renormalization effects, which can also lead to transient spectral change.^40^ However, as shown in Figure 3a, the FKO spectral shape shows negligible changes from early delay times (1–5 ps), when the free carriers are present in GaP, to later delay times (>100 ps), when the free carrier signal has decayed completely. This suggests that for the GaP/TiO_2_ sample, the TR spectral feature at the direct bandgap is dominated by the electric field-induced FKO signal caused by charge separation across the GaP/TiO_2_ interface.

The fit of the FKO spectra in Figure 3c allows the determination of the relative values of *E*_AC_ as a function of applied bias, assuming *E*_AC_ = −1 at −1.5 V. The determination of the absolute value of *E*_AC_ is not possible in this experiment because of the unknown proportionality constant that relates the FKO signal amplitude with the product of *E*_DC_*E*_AC_. *E*_DC_ can be calculated from the applied potential according to eq S5. The calculated *E*_DC_ and fitted relative *E*_AC_ is shown in Table 1. Fitting of the FKO spectra reveals that from 0 to −1.5 V, the FKO signal increases by 3.6 times because of the increase in both *E*_DC_ (by 1.4 times) and *E*_AC_ (by 2.5 times), with the latter indicating a 2.5-fold increase in the charge separation yield. This observation is consistent with the expected increase in charge extraction efficiency at a more negative applied bias, qualitatively similar to the charge separation efficiency increase observed under CW illumination in Figure 1c (∼2.2-fold increase from 0 to −1.5 V). As will be discussed below, more detailed analysis shows that *E*_DC_ values estimated using eq S5 is not accurate and should be corrected to obtain more reliable charge separation efficiencies.

**Table 1:** *E*_DC_ and *E*_AC_ of GaP/TiO_2_ Obtained from Fitting the Potential-Dependent FKO Spectra (Figure 3c) Measured at an Average 400 nm Excitation Power Density of 15 mW/cm^2^

applied bias (V)	–1.5	–1	–0.5	0
a*E*_DC_(kV/cm)	209	204	177	144
brelative *E*_AC_ (a.u.)	–1	–0.84	–0.57	–0.40

a Calculated according to eq S5.

b Obtained from fitting by eq S14. Only relative values of *E*_AC_ as a function of applied bias can be obtained in this work, and the relative *E*_AC_ value is set at −1 (a.u.) at −1.5 V. The negative sign indicates the opposite direction of *E*_AC_ and *E*_DC_.

The bias dependence of the TR kinetics of the free carrier and FKO signals is compared in Figure 3d. Under all applied potentials, the kinetics of the FKO signal growth agrees well with the free carrier signal decay kinetics. Furthermore, the growth time of the FKO signal shortens from ∼80 ps at 0 V to ∼5 ps at −1.5 V (see Table S2 and Figure S7). Thus, the bias-dependent TR kinetics reveal that at more negative applied potentials, the built-in electric field strength increases, which drives faster and more efficient separation of photogenerated electrons across the GaP/TiO_2_*p-n* junction.

**Figure 4:**
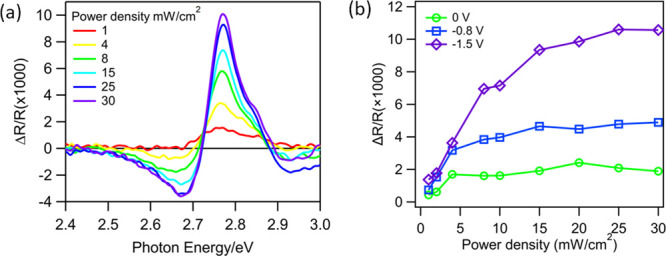
Transient reflectance spectra and kinetics of GaP/TiO_2_ measured as a function of average pulsed excitation power density. (a) Excitation power dependence of TR spectra and kinetics of GaP/TiO_2_ in the FKO signal region averaged at 100–200 ps at −1.5 V. (b) Transient FKO amplitude as a function of average excitation power densities measured at 0, −0.8, and −1.5 V, showing saturation of signal amplitude at higher fluences. The FKO amplitudes in (d) are monitored by subtracting 2.78 and 2.68 eV at a delay time of 50 ps, with ∼0.03 eV averaging spectral window around each point to reduce the noise.

To investigate the effect of the photogenerated carrier density on charge separation, transient reflectance spectra and kinetics were measured as a function of excitation fluence at fixed applied biases. The averaged TR FKO spectra at 100–200 ps and the transient FKO kinetics at 2.78 eV measured at −1.5 V for selected fluences are compared in Figure 4a and Figure S8, respectively. A comparison of the FKO spectral lineshape (Figure S8a) shows that they are independent of the excitation fluence, consistent with the weak field limit described above, and the FKO signal amplitude at long delay times saturates at large excitation fluence. This fluence dependence can be seen more clearly in Figure 4b, which shows the FKO signal amplitude at 2.78 eV and 50 ps saturates at high excitation fluence. At a certain potential, the saturation FKO signal amplitude indicates the maximum amount of charge separation, and a further increase in the excitation power yields no more charge separation. This suggests that at higher excitation power, losses due to charge recombination within the depletion region increase, which reduces the efficiency of charge transport across the *p-n* junction. This effect can be attributed to band flattening caused by a large transient carrier concentration.^41,42^ The band flattening effect originates from the high peak power of pulse laser excitation (SI S5), which introduces transient carrier density several orders larger than the dopant level. This undesirable effect can be mitigated by a larger initial band bending, as shown in Figure 4b: the −1.5 V curve saturates at ∼25 mW/cm^2^, while the 0 V curve saturates at 5 mW/cm^2^.

### Direct Measurement of Charge Separation Efficiency

The initial charge separation efficiency (ϕ_CS_), *i.e.*, the probability of transferring an electron across the GaP/TiO_2_ junction per absorbed photon, can be approximately determined from the FKO signal amplitude at 50 ps when the initial charge separation has reached its peak amplitude and slow interfacial charge recombination losses are small (Figure 3d). Because the FKO signal amplitude is proportional to the product of the *E*_AC_ (related to the amount of separated photogenerated electrons) and *E*_DC_, the charge separation efficiency (ϕ_CS_^′^) is proportional to the FKO amplitude at 50 ps after normalizing by the number of absorbed photons and *E*_DC_, according to eq 5.
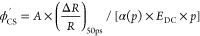
5

**Figure 5:**
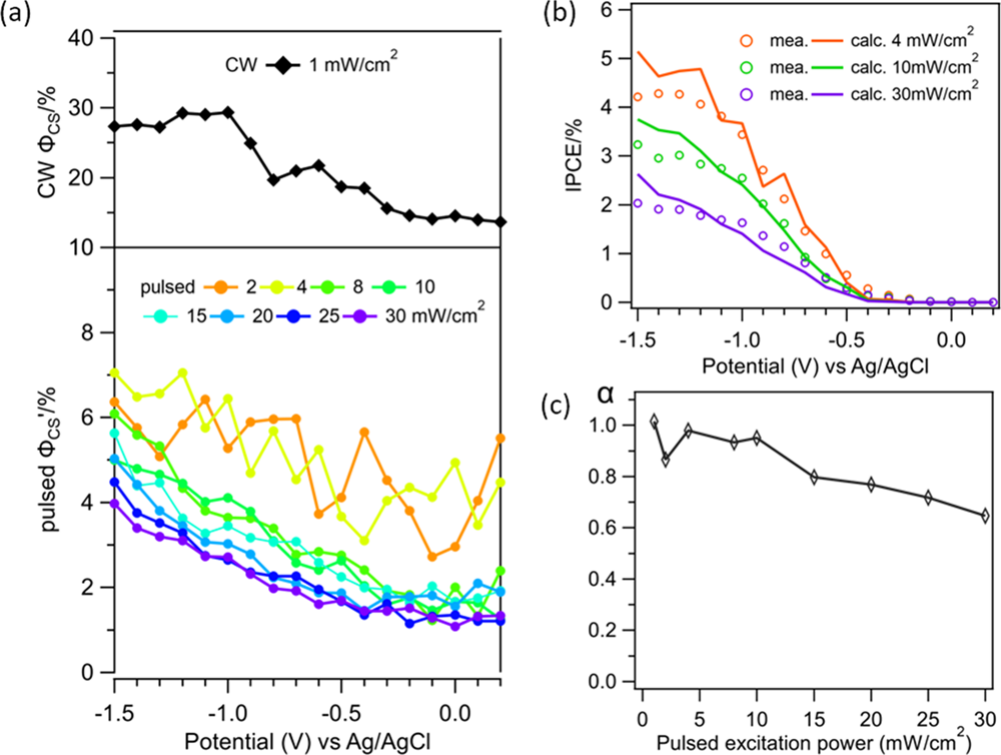
(a) Charge separation efficiency ϕ_CS_^′^ determined from the FKO signal amplitude at 50 ps according to eq 5. The CW ϕ_CS_ is obtained from Figure 1c. (b) Comparison of calculated IPCE and directly measured IPCE under pulsed excitation (left axis). The calculated IPCE is obtained by IPCE = ϕ_CS_^′^ϕ_red_ϕ_abs_, and the reduction efficiency ϕ_red_ is obtained from CW excitation in Figure 1d. (c) Plot of α (a factor accounting for reduction of DC electric field strength under transient reflectance conditions) as a function of excitation power density. The effective DC electric field coefficient (α) decreases from low to high excitation power due to increased charge accumulation.

In eq 5, *p* is the excitation power density (in mW/cm^2^), which scales with the number of absorbed photons,  is the FKO amplitude at 50 ps, *E*_DC_ is the DC field strength calculated from eq S5 (in kV/cm), α (≤1.0) is a coefficient that accounts for the decrease in DC field due to charge accumulation under transient reflectance measurement conditions (see below), and *A* (=1031 kV/cm·mW/cm^2^) is a scaling factor. The value of the scaling factor *A* is determined such that the calculated IPCE using ϕ_CS_^′^ according to eq 2 matches well with the measured IPCE at a low exciton power density (IPCE_calc._ = ϕ_abs_ϕ_CS_^′^ϕ_red_), where α is set to 1. The α value is allowed to change at higher excitation density to achieve the best match of calculated and measured IPCE values. In calculating IPCE, we have used the reduction efficiency ϕ_red_ obtained from CW excitation (Figure 1d), and the justification will be discussed below. Shown in Figure 5a is a plot of ϕ_CS_^′^ as a function of applied potential determined according to eq 5 at indicated excitation power densities. The comparison between the measured IPCE and the calculated IPCE using these ϕ_CS_^′^ values is shown in Figure 5b for selected excitation power densities, and the comparison for other power densities is shown in Figure S9b. The α values that were used for determining ϕ_CS_^′^ are plotted as a function of excitation power densities in Figure 5c.

As shown in Figure 5a, ϕ_CS_^′^ increases at more negative applied potentials, reaching its maximum value at the most negative bias of −1.5 V. A normalized comparison of ϕ_CS_^′^, shown in Figure S9a, shows that the bias dependences of ϕ_CS_^′^ measured at different excitation pulse densities are similar to each other and to those measured under CW excitations. It is important to note that the bias dependence of the directly measured ϕ_CS_^′^ is independent of the proportionality constant *A* or α used in eq 5, which only affects the absolute values of ϕ_CS_^′^. At a given applied potential, ϕ_CS_^′^ decreases at larger average pulsed excitation power densities and is significantly smaller than that measured under CW illumination. At −1.5 V, ϕ_CS_^′^ increases from 4% at 30 mW/cm^2^ to ∼7% at 4 mW/cm^2^ and this value increases up to ∼30% at CW illumination. This trend is consistent with the observation of FKO saturation at high pulse laser power shown in Figure 4d.

The measured IPCE under pulsed illumination continues to increase until an average pulsed excitation power density of 1 μW/cm^2^ (Figure 2d), while the IPCE values measured under a CW illumination range show negligible power dependence (Figure S4). This difference can be attributed to the much larger peak power and transient carrier density under the pulse excitation conditions. With the same average power density of 1 mW/cm^2^ (average carrier generation rate of 1.5 × 10^20^ cm^–3^ s^–1^ at 400 nm), the total number of generated carriers are the same under CW and pulsed illumination averaged over the repetition period of pulsed illumination (2 ms). Under CW illumination, the carriers are generated continuously, and the average photogenerated carrier density within the semiconductor can be estimated to be 1.5 × 10^10^ cm^–3^, assuming a transport time of 100 ps to sweep the carriers from the depletion region into TiO_2_ (see Figure 3d) and a pump penetration depth of 138 nm. This average photogenerated carrier density is much smaller than the dopant density of GaP (6.4 × 10^16^ cm^–3^) and has a negligible effect on the built-in electric field. Under pulsed illumination, one excitation pulse arrives at the sample every 2 ms and the carriers are generated within the 100 fs pulse width, corresponding to a transient carrier density of 2.9 × 10^17^ cm^–3^ in GaP, which is higher than its dopant density. As discussed in our previous study, the high transient carrier density under pulsed illumination leads to significant excitation power-dependent band flattening and recombination loss in GaP on the sub-picosecond time scale.^28^ Similar ultrafast recombination under pulsed illumination has also been reported in a previous transient optical study of Si nanowires and Si wafer.^43,44^ Thus, the reduced IPCE and ϕ_CS_^′^ under pulsed illumination can be attributed mainly to the enhanced charge recombination loss within the GaP during the initial separation stage caused by the large transient carrier density and band flattening effect.^42,45−49^ Because such loss is likely caused by an Auger recombination process, which depends on the concentrations of electrons (*n*) and holes (*p*) to the third power, *n*^2^*p* and *p*^2^*n*, the recombination loss under pulsed illumination increases at higher average power.^28,43,44^

On the time scale of catalysis (>2 ms), the average carrier density at the electrode surface is similar under similar average excitation power densities. For example, at −1.5 V and 1 mW/cm^2^ average power density, the photocurrent densities are 0.015 and 0.062 mA/cm^2^ under pulse and CW illumination, respectively (Figures S4 and S6). Because the steady-state photocurrent densities are dependent on the surface electron density, these results suggest that with the same average illumination power, the steady-state surface electron density is smaller under pulsed illumination conditions than CW, which is consistent with a lower initial charge separation efficiency under pulsed illumination. Furthermore, because the photoreduction quantum efficiency ϕ_red_ is determined by the competition of surface reaction and interfacial recombination at the steady state, it should be the same under CW or pulse illumination conditions, independent of illumination power.

As shown in Figure 5c, the α values used for determining ϕ_CS_′ decreases at higher excitation power densities, indicating a larger deviation of the DC field from that calculated from the applied bias according to eq S5. This can be attributed to the accumulation of photogenerated electrons in TiO_2_ under femtosecond laser excitation. In the TRS measurement, we compared the reflectance between the pump (light) and unpumped (dark) electrodes, and the time window between light and dark probe pulses (1 ms) is likely too short compared to the water reduction time. As a result, there is a possibility of accumulated electron in TiO_2_, and the GaP/TiO_2_ has not decayed to the true dark state for the “dark” probe signal. This is supported by the long photocurrent decay time (in the order of seconds) of the transient photocurrent measurement, as shown in Figure 1a. We hypothesize that such accumulation leads to a band flattening effect, which leads to *E*_DC_ values that are smaller than the true dark ones. Such accumulation effect increases with the average laser fluence, leading to the observed fluence-dependent α values shown in Figure 5c. Thus, accumulation of electrons in TiO_2_ not only increases the charge recombination loss but also decreases the initial charge separation efficiency. Future experiments at lower repetition rates and lower excitation powers may help to decrease this effect. Furthermore, simultaneous TRS and TPC measurements with the devices already under 1 sun CW illumination may provide a way to study these charge separation and water reduction steps under conditions more similar to device working conditions.^50^

## Discussion

### Key Loss Pathways

The results described above suggest that there are two main loss pathways in the overall photon-to-current conversion process in GaP/TiO_2_ electrodes: (1) the competition of interfacial electron transfer across the GaP/TiO_2_ junction with the electron–hole recombination within the GaP that limits the initial charge separation efficiency, ϕ_CS_; (2) the competition of water (proton) reduction by the transferred electrons in TiO_2_ and their interfacial charge recombination with the holes in the GaP limits the reduction quantum efficiency, ϕ_red_, as shown in Scheme 1. The first loss pathway can be overcome by increasing band bending, and the resulting built-in electric field through the applied bias. This is supported by the transient reflectance study (Figure 3c,d), which directly shows that at increasingly negative applied bias, both the rate and efficiency of the initial charge separation step increase. This is also consistent with the bias-dependent charge separation efficiency measured under CW and pulse illumination conditions (Figure 5a). Over the applied bias range from +0.2 to −1.5 V (vs Ag/AgCl), ϕ_CS_ increases at a more negative applied bias because the increased built-in field more effectively drives charge separation and suppresses charge recombination of photogenerated carriers within GaP. It is important to note that, at an applied bias of −1.5 V (vs Ag/AgCl) and CW illumination, although the ϕ_red_ reaches ∼100%, IPCE is still low (∼20%) because the main loss occurs at the initial charge separation process (ϕ_CS_ ∼ 30%). The charge separation efficiency further decreases to ∼4% at higher peak power under pulsed excitation conditions. For GaP/TiO_2_, the initial charge separation efficiency may be limited by the low extinction coefficient of GaP compared to other semiconductors, which results in a long optical absorption path. This leads to a large transport distance of the minority carriers to the interface, increasing the charge recombination losses within GaP.

The second recombination loss can be effectively suppressed by decreasing the interfacial recombination rate and/or increasing the catalytic reaction rate. As shown in Figure 1d, the reaction efficiency (ϕ_red_) increases rapidly at more negative potentials. ϕ_red_ is determined by the relative rates of interfacial reduction of proton and electron recombination with GaP valence band holes across the GaP/TiO_2_ junction. Because proton reduction involves electrons from the TiO_2_ conduction band or surface trap states, their energetics are independent of the applied potential on the GaP, which is dropped mainly within the GaP depletion region. As a result, we can assume that the proton reduction rate is independent of the potential and the observed potential dependence of ϕ_red_ reflects the change of the interfacial charge recombination rate. At less negative potentials, the extent of band bending is small and the concentration of the majority carrier (holes) at the surface is large, which increases the recombination loss. At potentials > −0.4 V, while IPCE approaches 0, ϕ_CS_ does not. This indicates that at this potential range, the GaP/TiO_2_ junction can still support initial charge separation, but the interfacial recombination of the separated electrons in TiO_2_ with the holes in GaP is much faster than the photoreduction reaction. At higher negative potentials (∼ −1.5 V), ϕ_red_ increases because the large band bending in GaP can slow down the recombination such that it is outcompeted by the slow proton reduction process on TiO_2_, even in the absence of catalyst layers.

It is interesting to note that even though the charge separation efficiency under femtosecond pulsed excitation conditions is small, ∼1–7% as shown in Figure 5a, TR spectroscopy can provide a sensitive probe of the charge separation kinetics and its dependence on the applied bias. This can be attributed to the small penetration depth of the reflectance probe and the ability to selectively probe the near surface FKO signal that is directly correlated to the separated charge carriers across the GaP/TiO_2_ interface. This feature overcomes the technical challenge of transient absorption measurements in which all carriers are probed and it is difficult to isolate the small population of bias-dependent separated carriers from the total carrier population. Furthermore, because the transient reflectance technique does not require optically transmissive electrodes, it can be applied to a wider range of photoelectrodes under device operation conditions. Because of these advantages, we believe that transient reflectance spectroscopy is a useful technique for *operando* study of charge carrier dynamics of photoelectrodes. Although our initial study is focused on GaP/TiO_2_, it can be extended to full photoelectrode systems with surface-attached catalysts, such as a semiconductor/TiO_2_/Pt photoelectrode, with better HER performance, as long as the surface catalyst does not block light penetration into the light-absorbing semiconductor. We will explore this capability in future work.

## Conclusions

In summary, we report simultaneous *in situ* transient reflectance spectroscopy and transient photocurrent measurements of photoelectrochemical cells with TiO_2_-protected GaP photocathodes for water reduction. TR spectroscopy enables the direct probe of the kinetics of the free carriers inside the GaP and separated carriers across the GaP/TiO_2_ junction. Comparison of the bias- and excitation fluence-dependent carrier kinetics and IPCE enables the direct measurement of the initial charge separation efficiencies ϕ_CS_ and provides support of a transient photocurrent model that decomposes the overall IPCE into the product of the quantum efficiencies of three stages (IPCE = ϕ_abs_ϕ_CS_ϕ_red_). According to this model, there are two key loss pathways in the overall light-to-photocurrent conversion process: first, charge recombination within the GaP that competes with the initial built-in field-driven charge separation across the GaP/TiO_2_ junction to reduce the initial charge separation efficiency (ϕ_CS_), and second, the interfacial recombination of the separated electrons in TiO_2_ with the holes in the GaP that competes with proton reduction, limiting the photoreduction quantum efficiency (ϕ_red_). Our results show that both loss pathways can be reduced at a more negative bias to increase the built-in electric field that facilitates charge separation and suppresses recombination. However, at an applied bias of −1.5 V (vs Ag/AgCl) and CW illumination, although the ϕ_red_ reaches ∼100%, the initial charge separation process ϕ_CS_ is ∼30%, which limits the overall IPCE to ∼20%. Our study gives a detailed, sequential time-resolved view of charge carriers dynamics from generation, separation, and recombination to reaction in a model photoelectrochemical system and provides helpful insights into key efficiency limiting factors.

## Supporting Information Available

Photocurrent and IPCE under CW and pulsed illumination, FKO fitting and kinetics fitting, and ϕ_CS_^′^ calculation (PDF)
