# Supplementary material for: Direct In Situ Measurement of Quantum Efficiencies of Charge Separation and Proton Reduction at TiO2-Protected GaP Photocathodes
Source: J Am Chem Soc. 2023 Jan 30;145(5):2860–9. doi: 10.1021/jacs.2c10578 (PMC9912250; doi:10.1021/jacs.2c10578)
Supplement: Supplementary file 1 — ja2c10578_si_001.pdf [file ja2c10578_si_001.pdf]

Supporting Information

Direct *in-situ* Measurement of Quantum Efficiencies of Charge-Separation and Proton Reduction at TiO_2 protected GaP Photocathodes

Zihao Xu[1,2], Bingya Hou[3], Fengyi Zhao[1], Sa Suo[1], Yawei Liu[1], Haotian Shi[4], Zhi Cai[3], Craig L. Hill[1], Djamaladdin G. Musaev[1,5], Matthew Mecklenburg[6], Stephen B. Cronin[3,4*], and Tianquan Lian[1*]

1. Department of Chemistry, Emory University, 1515 Dickey Dr, Atlanta, GA, 30322, USA
2. ZJU-Hangzhou Global Scientific and Technological Innovation Center, Zhejiang University, Hangzhou, 310014, China.
3. Department of Electrical Engineering, University of South California, 3710 McClintock Ave, Los Angeles, CA, 90089, USA
4. Department of Chemistry, University of South California, 3710 McClintock Ave, Los Angeles, CA, 90089, USA
5. Cherry L. Emerson Centre for Scientific Computation, Emory University, 1515 Dickey Drive, Atlanta, GA, 30322, USA
6. Core Center of Excellence in Nano Imaging (CNI), University of South California, 814 Bloom Walk, Los Angeles, CA, 90089, USA.

Table of Contents

S1 Materials Preparation 2

S2 Photoelectrochemical Setup 5

S3 Mott-Schottky analysis of GaP/5-nm TiO_2 electrode 6
S4 IPCE under CW illumination....7
S4.1 GaP/TiO_2 reflection loss in PEC cell. 8
S5 IPCE under femtosecond pulsed illumination. 9
S6 Transient Reflectance Spectroscopy....10
S6.1 Penetration depth of GaP (100) single crystal....11
S6.2 FKO fitting function....11
S6.3 Power and bias dependence of TR signal amplitudes and kinetics....14
S6.4 Relative charge separation efficiency calculation....18
References....19

S1 Materials Preparation

The Zn-doped 100-oriented GaP single crystals have a dopant concentration of ~6.4×10^{16} cm^{-3} (determined by impedance spectroscopy, see below and Fig S1 for detail). The electrode was prepared according to a previous procedure.[1-2] In brief, the TiO_2 layer was deposited by atomic layer deposition (ALD) at 250 °C with $TiCl_4$ as titanium source and water vapor as oxygen source. The ALD TiO_2 was verified to be of anatase phase with Raman spectroscopy in previous study.[3] A Ga-In alloy was painted to the back of the GaP for ohmic contact which is later connected to the copper conductive tape and sealed with epoxy to form an electrode for study.

The ALD TiO_2 protected GaP demonstrated excellent stability under CO_2 reduction conditions (>8h measured) while enhancing onset potential and photocurrent.[1-2] A previous photoelectrochemical study of ALD TiO_2 protected *p*-GaP photocathodes reported that the light-induced hydrogen evolution reaction (HER) performance depends sensitively on the thickness of the TiO_2 layer.[1-2] The onset potential shifts to less negative values, and the photocurrent at the same potential increases with the ALD thickness, reaching the best performance at ~10 nm; further increase of the ALD thickness decreased the performance. To understand the enhancement mechanism produced by the ALD TiO_2 layer, we have chosen GaP electrodes with 5 nm TiO_2 layer for the simultaneous *in-situ* transient reflection spectroscopy and photoelectrochemical measurements described in the main text. Two batches of GaP/TiO_2 electrodes were used in this study and they produce similar IPCE performance under CW illumination.

Electron Microscopy: Cross-sectional high-resolution transmission electron microscope (HRTEM) images of the GaP/TiO_2 interface are shown in Figure S1. Here, the TiO_2 film was deposited by atomic layer deposition (ALD). A thick layer of Pt was deposited on the GaP/TiO_2 in order to provide structural stability during the TEM cross-section sample preparation, however, this Pt layer was not used in the photoelectrochemical measurements. These figures show a TEM cross-sectional image of GaP with a 5 nm thickness (nominal thickness) layer of amorphous TiO_2. This corresponds to 100 cycles in the ALD process. From TEM images, we find that the actual thickness is 4.95 nm, in good agreement with the nominal thickness value. While the TiO_2 appears to be predominantly amorphous, some crystal lattice features can be seen near the gallium phosphide interface.

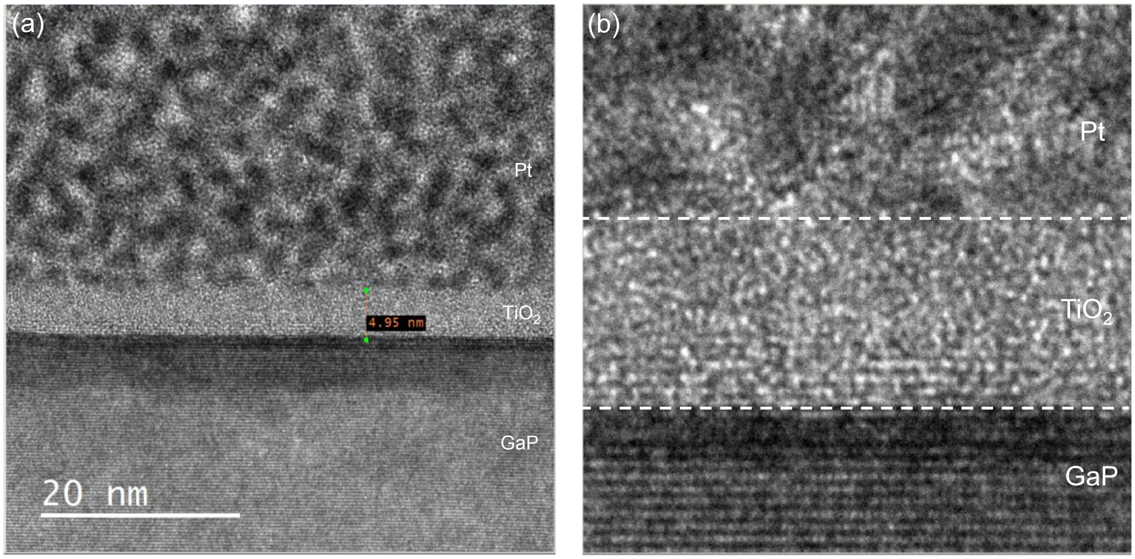

Figure S1. Cross-sectional transmission electron microscopy images of the GaP/TiO_2 interface. Here, the sample is capped with a thick layer of platinum, not used in the photoelectrochemical measurements.

In Figure S2, energy-dispersive X-ray spectroscopy (EDS) was used to spatially map the elemental composition of the sample. Figures S2d and S2g shows the spatial maps corresponding to oxygen and titanium, respectively. Similarly, Figures S2c and S2e show the spatial maps corresponding to gallium and phosphorus, respectively. These spatial maps confirm the compositional labeling and interfacial boundaries drawn in Figure S1.

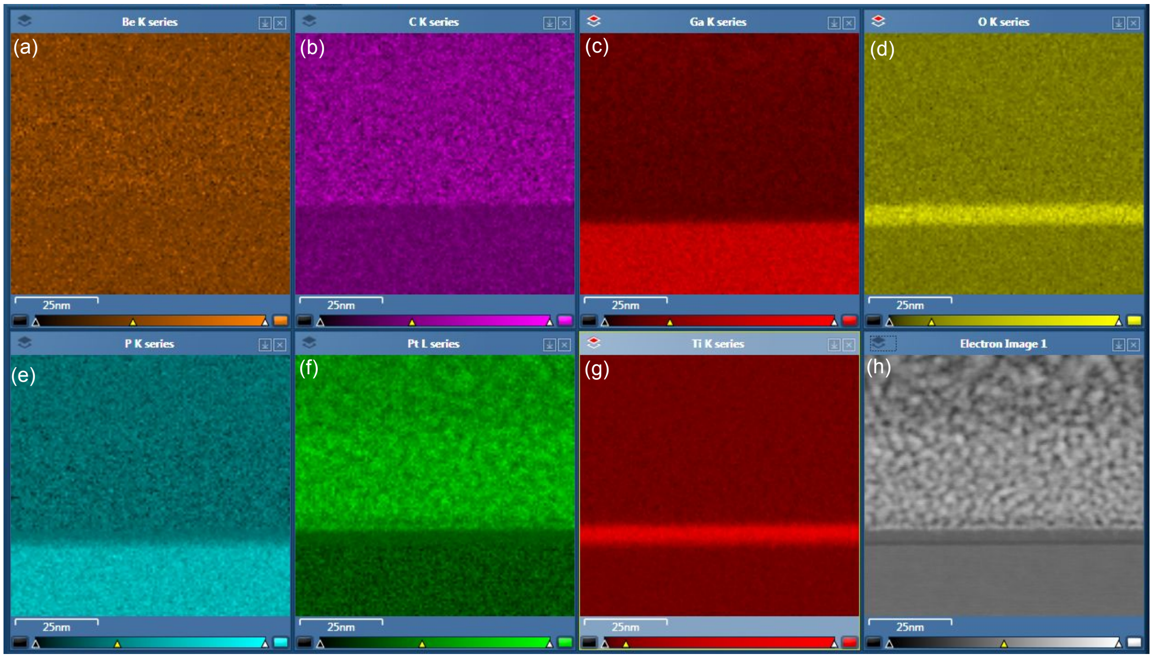

Figure S2. Energy-dispersive X-ray spectroscopy (EDS) images of the GaP/TiO_2 interface collected in the scanning transmission electron microscopy (STEM) mode. Here, the sample is capped with a thick layer of platinum, not used in the photoelectrochemical measurements.

S2 Photoelectrochemical Setup

All electrochemical experiments were carried out by a CHI660E workstation (CH Instrument) in a three-electrode setup. A Pt wire acts as a counter electrode and Ag/AgCl (in 1M KCl) acts as a reference electrode (CH Instrument). The electrolyte is a buffered aqueous solution at pH=7 with ionic strength of 0.2 M purged with N_2 prior to the experiment, which is prepared by dissolving 96 mg disodium phosphate, 156 mg monosodium phosphate and 270 mg sodium sulfate into 100 mL water under room temperature. All chemicals used were purchased from Sigma-Aldrich without further preparation. All potential is relative to Ag/AgCl unless stated otherwise. The CW illumination experiment used a 405 nm LED (Ocean Optics) coupled with a focal lens and a ND filter to adjust the beam size and intensity.

S3 Mott-Schottky analysis of GaP/5-nm TiO_2 electrode

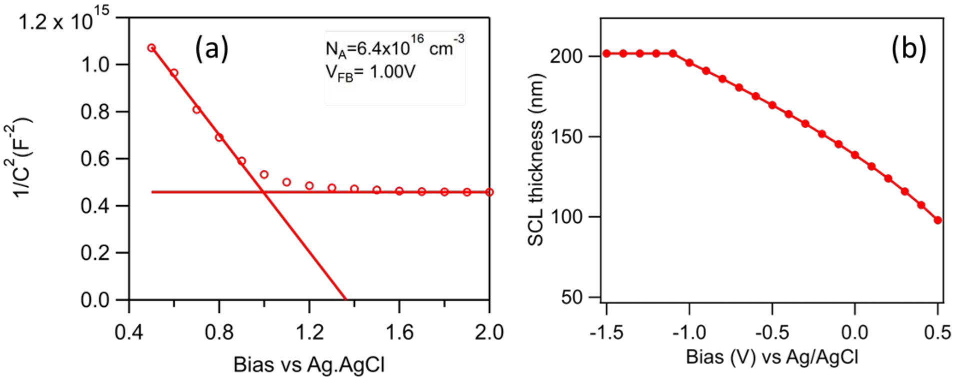

Figure S3. (a) Mott-Schottky analysis of GaP/5nm-TiO_2 electrode: flat band potential (1.00 V vs Ag/AgCl) and dopant concentration ($6.4*10^{16}$ cm^{-3}) measured by impedance spectroscopy in the dark. (b) Space charge layer (SCL) thickness under different bias calculated from Eq. S3. The SCL stops increasing when the bias applied is more negative than the conduction band edge (-1.12 V vs Ag/AgCl).

The dopant concentration is determined by Mott-Schottky equation:

$$C^{-2} = \frac{2}{qA^2\varepsilon N_A}\left(V - V_{fb} - \frac{k_B T}{q}\right) \quad \text{S1}$$

Here, C is the capacitance, q is the elementary charge, ε is the permittivity of GaP, V is applied potential, V_{fb} is the flat band potential, k_B is Boltzmann constant and N_A is the dopant concentration. The capacitance of the GaP/TiO_2 electrode is measured as a function of applied potential by impedance spectroscopy (Figure S3). By fitting the capacitance-bias data in Figure S3 to Equation S1, the dopant concentration can be determined to be $6.4*10^{16}$ cm^{-3} with 0.16 cm^2 electrode surface area (4 mm by 4 mm). From the intercept of the fit to constant capacitance as shown in Figure S3, the flat band potential is calculated to be 1.00 V (*vs* Ag/AgCl) according to:

$$V_{fb} = V_x - \frac{k_B T}{q} \quad \text{S2}$$

Using the values of dopant density and the flat band potential determined above, the depletion width in GaP as a function of applied potential can be calculated:

$$x_p = \sqrt{\frac{2\varepsilon}{q}\frac{1}{N_A}|V - V_{fb}|} \quad \text{S3}$$

The electrical field could be described by:

$$E(x) = \frac{qN_A}{\varepsilon}x, 0 < x < x_p \qquad \text{S4}$$

The electrical field at the interface is calculated by:

$$E = \frac{qN_A}{\varepsilon}x_p = \frac{qN_A}{\varepsilon}\sqrt{\frac{2\varepsilon}{q}\frac{1}{N_A}|V - V_{fb}|} = \sqrt{\frac{2qN_A}{\varepsilon}|V - V_{fb}|} \quad \text{S5}$$

The M-S measurement in our study indicates the flat band potential is 1.00V vs Ag/AgCl (in 1M KCl), then the valence band edge can be calculated to be 1.14 V vs Ag/AgCl, which is 1.36 V vs NHE for an intrinsic carrier density (n_i) of 2 cm^{-3}, bandgap of 2.26 eV, and a dopant density of $6.4x10^{16}$ cm^{-3}.[4] This matches well with literature values of 1.25 V vs NHE.[5-6] When the applied bias approaches the conduction band edge, the electrical field strength will reach its maximum (209 kV/cm) at -1.1 V vs Ag/AgCl.

S4 IPCE under CW illumination

Figure S4a shows the IPCE as a function of applied bias at several illumination power densities, and Figure S4b shows a pseudo-color map of the IPCE as a function of illumination power density and applied bias under CW LED light illumination. The IPCE increases at more negative applied bias until -1.0 V, after which it reaches a plateau value of ~17%. These potential-dependent IPCE values show a negligible dependence on the CW illumination power density from 0.04 to 0.12 mW/cm^2.

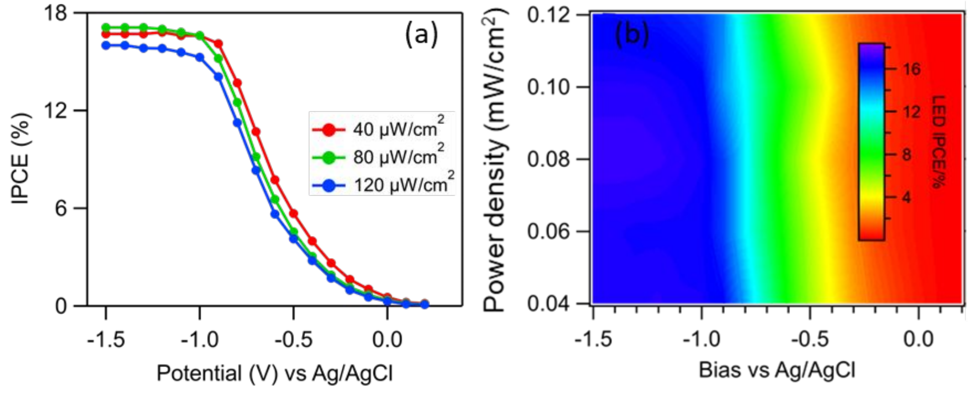

Figure S4. IPCE of GaP/TiO_2 under CW LED light illumination at 405 nm. (a) IPCE as a function of applied bias at indicated power densities and (b) 2D pseudo-color map of IPCE as a function of applied bias and excitation power density.

S4.1 GaP/TiO_2 reflection loss in PEC cell.

The reflection loss of the GaP/TiO_2 electrode is defined as the ratio of the reflected beam power and the input beam power. The measurement was done in the PEC cell, where both the air/cell interface and water/electrode interface produce a reflection. The reflection loss, as shown in Figure S5, is 6.6% for air/cell reflection and 18.4% for water/electrode interface. In total, the reflection loss is 25%. Thus, 75% incident photon is absorbed by the GaP/TiO_2.

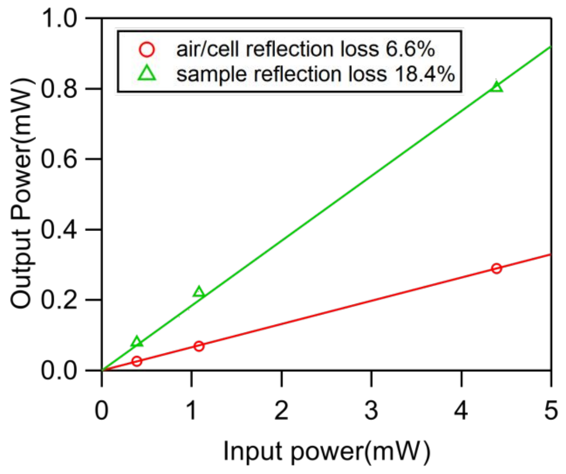

Figure S5. The reflection loss of the air/cell interface (red circle) and water/electrode interface (green triangle) is measured by the slope of the output power to the input power.

S5 IPCE under femtosecond pulsed illumination.

The typical photocurrent transient under pulse laser illumination are shown in Figure S6a. The temporal resolution of the electrochemical workstation is not able to resolve individual laser pulses, thus, the measured photocurrent is an average of individual pulse induced photocurrent. The stable photocurrent $j(\infty)$ is obtained similarly to the transient photocurrent in Scheme 1 and Figure 1a, by the difference between light on and light off as shown in Figure S6a. Measured IPCEs as a function of excitation power density and applied potentials under pulsed femtosecond illumination are shown as a pseudo-color plot in Figure S6b. Because of the short laser pulse (150 fs) and low repetition rate (500 Hz) used for the transient reflectance measurement, the peak power is $\sim 6 \times 10^{10}$ times higher than the average power and both are indicated in Figure S6b. At a given illumination power density, IPCE increases at more negative applied biases; and at a fixed applied bias, the IPCE decreases at higher excitation power densities. The highest IPCE is 4.6% at -1.5V under 400 nm pulsed laser excitation with an average excitation power density of 1 mW/cm^2, significantly smaller than those measured under CW illumination. Lowering the fs pulsed illumination power can increase the IPCE comparable to that under CW illumination as shown in Figure 2d, but such power is insufficient for the transient reflectance spectroscopic measurement.

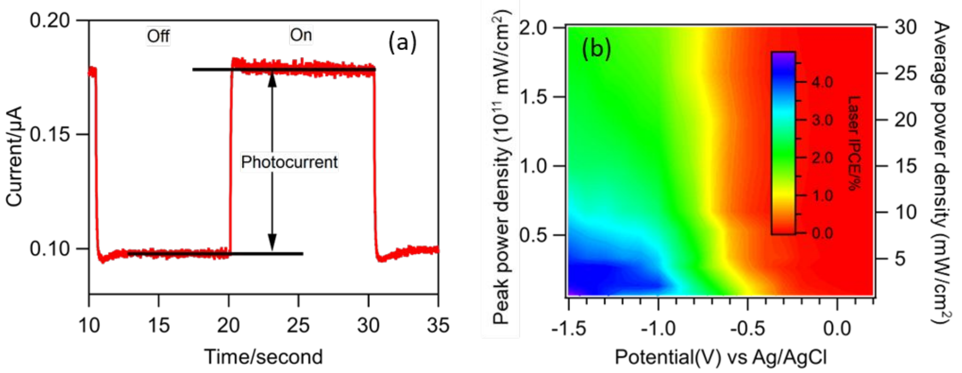

Figure S6. IPCE of GaP/TiO_2 under pulsed femtosecond laser illumination at 400 nm. (a) Current as function of time with modulated (on/off) pulse laser excitation light under -1 V and power density of 10 mW/cm^2. Photocurrent is calculated from the difference between the steady state light on and off current values at plateau regions indicated on the figure to avoid contributions of non-faradaic (charging and discharging) current. (b) 2D pseudo-color map of IPCE as a function of applied bias and average excitation power density, measured with pulse 400 nm illumination.

S6 Transient Reflectance Spectroscopy

Femtosecond transient reflectance spectroscopy (TRS) setup consists of a Coherent Legend system which produces 150 fs 800 nm pulses at kilohertz with a pulse energy of 2.3 mJ. Pump pulses at 400 nm (photon energy 3.1 eV) are generated by converting a portion of 800 nm pulses by second harmonic generation in a type I BBO crystal. The pump pulse is chopped at 500 Hz to provide pumped and un-pumped contrast. Pump beam size at the sample is circular with an area of ~1×10^{-3} cm^2. A fraction of 800 nm is focused onto a constantly rotating CaF_2 crystal to generate a white light probe pulse from 1.5 eV to 3.1 eV. The delay time between pump and probe pulses is achieved by delaying the pump pulse through a delay stage. The probe light was divided by a beam splitter to provide a signal which interacts with the sample and a reference which is used to compensate noise and fluctuation. The angle of incidence (AOI) for pump pulse is 35 degrees while probe light

at 45 degrees. The probe and reference beams were collected by fibers and guided into grating and CMOS detector (Ultrafast System, Helios).

S6.1 Penetration depth of GaP (100) single crystal

Ellipsometry data from previous study for bare GaP (100) single crystals are used to estimate the penetration depth of the pump and probe beams.[7] The pump penetration depths is estimated to be 138 nm at 3.1 eV by $d=1/\alpha=\lambda/(4\pi\kappa)$, and the probe detection depth is 8~21 nm for 3.1~1.5 eV by $d=1/\alpha=\lambda/(4\pi n)$, where n and k are the real and imaginary part of the refractive index.

S6.2 FKO fitting function

According to the Gauss law, the field strength at the GaP/TiO_2 interface is given by,

$$(E_{AC})_{x=0} = \frac{\sigma}{\varepsilon\varepsilon_0} \qquad \text{S6}$$

In Equation S6, σ is the total transferred charge across the GaP/TiO_2 interface per unit GaP surface area, ε is the relative permittivity of GaP and ε_0 is vacuum permittivity. Thus, the FKO-induced ΔR/R signal amplitude is linearly proportional to the concentration of electrons that are transferred into TiO_2, providing a convenient probe of the interfacial charge separation in the GaP/TiO_2 *p-n* junction.[8]

In the weak field and large broadening energy limit, the FKO spectrum can be simplified to a third derivative form.[9-11] This condition applies to the GaP/5-nm TiO_2 system we studied, because the sequential oscillation of FKO signal is heavily damped in the high energy probe region (>2.8 eV) in Figure 3. The third derivative form under a constant DC field is:[9-12]

$$\frac{\Delta R}{R} \propto (\hbar\theta)_j^3 Re[e^{i\phi_j}(E - E_{gj} + i\Gamma_j)^{-m}] \quad \text{S7}$$

Here, two transitions (*j*=1,2) corresponding for heavy hole and light hole are necessary to describe the FKO signals. Φ is the phase difference between the real and imaginary part of the refractive index, E_g is the band gap, Γ is the broadening energy, m is 2.5 for a direct transition critical point in GaP. In Equation S7,

$$(\hbar\theta)^3 = \frac{e^2\hbar^2 {E_{DC}}^2}{2\mu} \quad \text{S8}$$

where μ is the effective mass defined for heavy hole and light hole in GaP:[4]

$$\mu_{hh} = \frac{m_e m_h}{m_e + m_h} = 0.08 m_0 \quad \text{S9}$$

$$\mu_{lh} = \frac{m_e m_h}{m_e + m_h} = 0.055 m_0 \quad \text{S10}$$

Upon light excitation:

$$(\hbar\theta_L)^3 = \frac{e^2\hbar^2 (E_{AC} + E_{DC})^2}{2\mu} \quad \text{S11}$$

therefore, the light-modulated FKO signal can be described as:

$$\frac{\Delta R}{R} \propto \sum_{j=1}^{2} \frac{e^2\hbar^2[(E_{AC} + E_{DC})^2 - {E_{DC}}^2]}{2\mu_j} Re\left[e^{i\phi_j}(E - E_{gj} + i\Gamma_j)^{-m}\right] \quad \text{S12}$$

when E_{AC} is small, $(E_{AC} + E_{DC})^2 - {E_{DC}}^2 \approx 2E_{AC}E_{DC}$, then the above equation can be approximated to:

$$\frac{\Delta R}{R} \propto \sum_{j=1}^{2} \frac{e^2\hbar^2 E_{AC} E_{DC}}{\mu_j} Re\left[e^{i\phi_j}(E - E_{gj} + i\Gamma_j)^{-m}\right] \quad \text{S13}$$

which can be written as Equation S14 for fitting:

$$\frac{\Delta R}{R} = \sum_{j=1}^{2} A_j \frac{e^2\hbar^2}{\mu_j} E_{AC} E_{DC} Re\left[e^{i\phi_j}(E - E_{gj} + i\Gamma_j)^{-m}\right] \quad \text{S14}$$

A_j is an arbitrary coefficient to adjust the amplitude.

Combining Equation S14 and Equation S6 leads to the conclusion that the FKO amplitude is proportional to the amount of charge carriers that are separated across the junction under a given E_{DC}. For a specific sample, such as GaP/5-nm TiO_2, only E_{DC} and E_{AC} change with applied bias and power and all other fitting parameters are material-specific constants. The fitting parameters are shown in Table S1. The determination of the absolute value of E_{AC} is not possible in this experiment, because of the unknown proportionality constants (A_j) that relate the FKO signal amplitude with the product of E_{DC} E_{AC}. E_{DC} as a function of applied bias can be calculated using Equation S5. The fit allows us to determine the relative values of E_{AC}. Therefore, we set the E_{AC} value to be -1 at -1.5V and the FKO spectra at all potentials are fit globally with potential dependent E_{AC} and potential independent parameters (A_1, Eg_j, ϕ_j, Γ_j). The universal fitting parameters (same for different bias) for Figure 3c is listed in Table S1 and the values for E_{DC} and E_{AC} are listed in Table 1.

Table S1. Fitting parameters for Figure 3c

**Fitting parameter**	**GaP/5-nm TiO_2**
Eg_1/eV	2.765±0.002
Eg_2/eV	2.821±0.007
A_1	-0.0016
A_2	-0.00023
ϕ_1	3.67±0.08
ϕ_2	2.53±0.39
Γ_1/meV	84.2±2.2
Γ_2/meV	62.0±6.5

S6.3 Power and bias dependence of TR signal amplitudes and kinetics

The kinetics shown in Figure 3b and 3d are fitted by a multiexponential model, the ΔR/R kinetics can be described as:

$$\frac{\Delta R}{R} = \sum_i a_i e^{-t/\tau_i} \qquad \text{S15}$$

Here, for FKO growth kinetics there are two growth components, one decay component and a long-lived constant amplitude, and for free carrier kinetics there are two decay components. The two growth time constants of FKO kinetics correspond to the decay time constants of the free carrier. In the fitting, these constants are linked. The fitting function is then convoluted with the instrument response function (IRF) with a time constant τ_{IRF}. The FKO kinetics and free carrier kinetics under various biases can be fitted with Equation S15. The slow growth corresponding time constant is the τ_1 in Table S2, this time constant decreases with the more negative biases. The amplitude (a_0~a_3) in Table S2 takes the unit of ΔR/R(*1000).

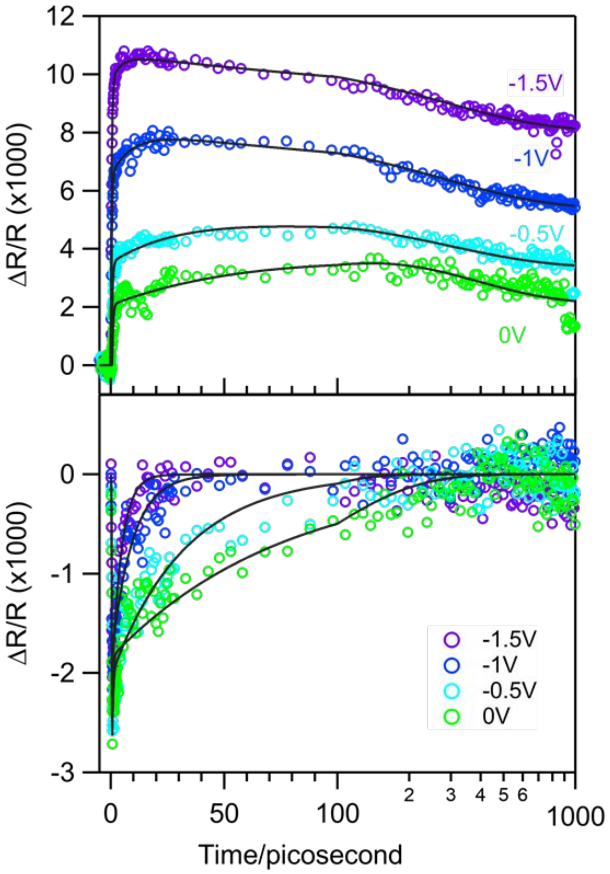

Figure S7. Multiexponential fit of FKO and free carrier kinetics under 15 mW/cm^2 400 nm excitation density. The FKO kinetics data is the same as Figure 3d. The fitting parameters are listed below.

Table S2. Fitting parameter for the FKO kinetics growth under various applied bias.

	-1.5 V		-1.0 V		-0.5 V		0 V	
	**FKO**	**Free**	**FKO**	**Free**	**FKO**	**Free**	**FKO**	**Free**
τ_{IRF}/ps	0.183							
$^1a_0$	-9.71±0.11	-0.09±0.3	-6.51±0.08	-0.68±0.28	-3.53±0.05	-1.19±0.25	-2.08±0.04	-1.38±0.24
τ_0/ps	0.40±0.02							
a_1	-0.96±0.14	-2.7±0.1	-1.56±0.10	-1.81±0.08	-1.90±0.11	-2.01±0.05	-3.10±0.22	-1.84±0.04
$^2\tau_1$/ps	4.34±0.51		9.25±0.82		32.6±2.3		76.2±5.3	
a_2	2.62±0.06	-	2.68±0.07	-	2.07±0.11	-	3.07±0.23	-
$^3\tau_2$/ps	287±15	-	287±15	-	287±15	-	287±15	-
$^4a_3$	8.05	-	5.39	-	3.36	-	2.11	-

[1]a_0 accounts for the fast growth of FKO, and the corresponding fast decay of free carrier. [2]τ_1 is the time constant of the slow growth, which shows a clear bias dependence. [3]The decay of FKO signal is due to the carrier's lateral diffusion, which we will further explain in a future study. We expect this lateral diffusion to be independent of bias, thus τ_2 is the same for all the biases. [4]a_3 is the FKO long lived constant amplitude, which is calculated by $-(a_0+a_1+a_2)$.

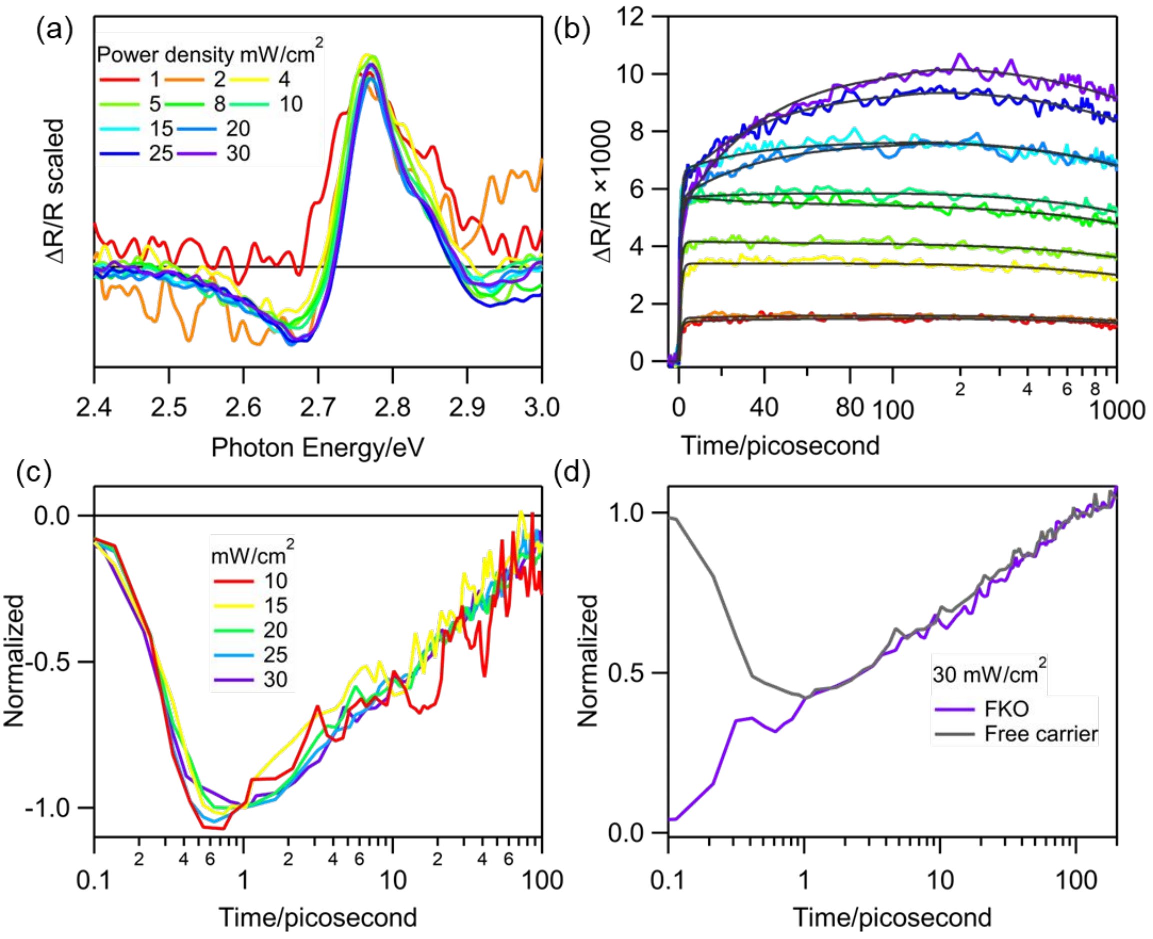

Figure S8. (a) Normalized power dependence FKO spectra at -1.5 V and various excitation power. Spectra averaged at 100-200 ps delay time. (b) FKO kinetics with the same color legends, obtained from 2.78-2.68 eV, fitted by Equation S15. (c) Free carrier kinetics at 1.5 eV after normalization. (d) Comparison of the slow growth component kinetics of FKO and free carrier.

The growth kinetics of the FKO signal also depend on the excitation fluence, as shown in Figure S8b. At low fluence, the FKO kinetics are dominated by the fast component within 1 ps (τ_1=0.57 ps, see fitting in Figure S8b and Table S3) with only a minor contribution from the slow growth component (τ_2=39.0 ps) on the 1-100 ps time scale. After reaching the saturation fluence, the amplitude of the fast component decreases from 90% to 50% and the slow component increases from 10% to 50%, respectively, while the time constants for these components remain power-independent. We attribute the fast growth component to the electrons in the depletion region of GaP, which is driven to the TiO_2 by the built-in field, and the slow component to carriers within the bulk that undergo slower diffusive transport. The fast component is too fast to be captured in the free carrier decay kinetics, but the slow growth component agrees well with the free carrier decay, as shown in Figure S8d. The result suggests that at low fluence, the charge separation across the GaP/TiO_2 *p-n* junction is driven by the built-in field. As the excitation power increases, the higher carrier density screens the built-in electric field in the depletion region (also known as band flattening) and increases the recombination loss of photogenerated charge carriers, giving rise to the saturation behavior at high fluences.[13-16]

The fast and slow growths of the FKO signal are fitted by Equation S15 with the same time constant and with different amplitudes. The fitting parameters are listed in Table S3 below. The fast component ratio decreases from ~90% to ~50% as the power increases.

Table S3. Fitting of FKO growth kinetics in Figure S8b

Power	a_1/%	a_2/%	τ_1/ps	τ_2/ps	τ_3/ns
1	89±33	11±4	0.57±0.01	39.0±1.3	6.5±0.11
2	85±15	15±3			
4	94±24	6±2			
5	82±32	18±7			
8	73±23	27±8			
10	65±19	35±10			
15	51±5	48±5			
20	57±2	43±2			
25	57±2	43±1			
30	54±1	46±1			

S6.4 Relative charge separation efficiency calculation

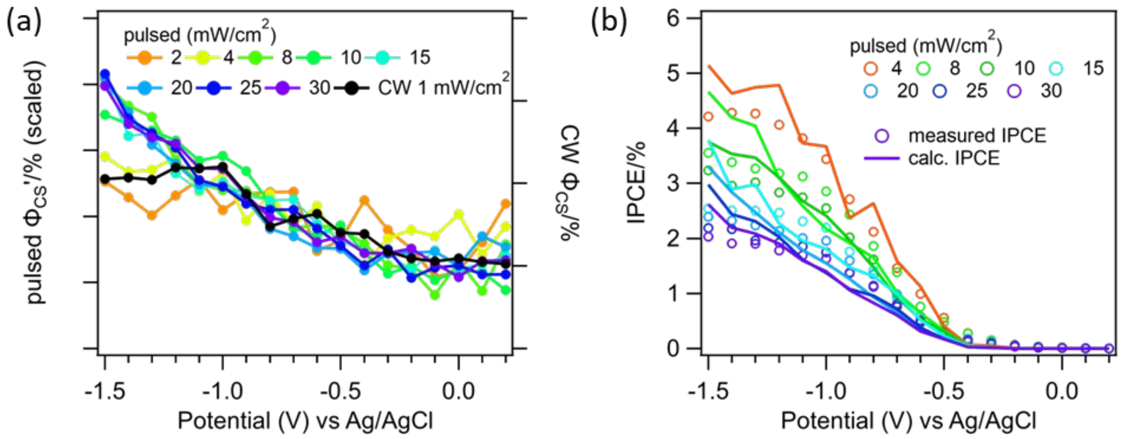

Figure S9. (a) Scaled ϕ_{CS}' under pulsed condition and comparison with ϕ_{CS} under CW condition. (b) Comparison between measured and calculated IPCE obtained by the same method in Figure 5b.

References

1. Zeng, G.; Qiu, J.; Li, Z.; Pavaskar, P.; Cronin, S. B., Co2 Reduction to Methanol on Tio2-Passivated Gap Photocatalysts. *ACS Catalysis* **2014,** *4* (10), 3512-3516.
2. Qiu, J.; Zeng, G.; Pavaskar, P.; Li, Z.; Cronin, S. B., Plasmon-Enhanced Water Splitting on Tio2-Passivated Gap Photocatalysts. *Physical Chemistry Chemical Physics* **2014,** *16* (7), 3115-3121.
3. Qiu, J.; Zeng, G.; Ha, M.-A.; Ge, M.; Lin, Y.; Hettick, M.; Hou, B.; Alexandrova, A. N.; Javey, A.; Cronin, S. B., Artificial Photosynthesis on Tio2-Passivated Inp Nanopillars. *Nano Letters* **2015,** *15* (9), 6177-6181.
4. Levinshtein, M., *Handbook Series on Semiconductor Parameters*. World Scientific: 1997; Vol. 1.
5. Nozik, A. J.; Memming, R., Physical Chemistry of Semiconductor−Liquid Interfaces. *The Journal of Physical Chemistry* **1996,** *100* (31), 13061-13078.
6. Nozik, A. J., Photoelectrochemistry: Applications to Solar Energy Conversion. *Annual Review of Physical Chemistry* **1978,** *29* (1), 189-222.
7. Jellison, G. E., Optical Functions of Gaas, Gap, and Ge Determined by Two-Channel Polarization Modulation Ellipsometry. *Optical Materials* **1992,** *1* (3), 151-160.
8. Xu, Z.; Hou, B.; Zhao, F.; Cai, Z.; Shi, H.; Liu, Y.; Hill, C. L.; Musaev, D. G.; Mecklenburg, M.; Cronin, S. B.; Lian, T., Nanoscale Tio2 Protection Layer Enhances the Built-in Field and Charge Separation Performance of Gap Photoelectrodes. *Nano Letters* **2021**.
9. Shen, H.; Dutta, M., Franz–Keldysh Oscillations in Modulation Spectroscopy. *Journal of Applied Physics* **1995,** *78* (4), 2151-2176.
10. Shen, H.; Pollak, F. H., Generalized Franz-Keldysh Theory of Electromodulation. *Physical Review B* **1990,** *42* (11), 7097.
11. Hall, D. J.; Hosea, T. J. C.; Lancefield, D.; T., T. W.; J., L. A. F.; G., B. E., Airy Function Analysis of Franz–Keldysh Oscillations in the Photoreflectance Spectra of In1−Xgaxasyp1−Y Layers. *Journal of Applied Physics* **1997,** *82* (6), 3092-3099.
12. Pollak, F. H., Study of Semiconductor Surfaces and Interfaces Using Electromodulation. *Surface and Interface Analysis* **2001,** *31* (10), 938-953.
13. Lantz, J. M.; Corn, R. M., Time-Resolved Optical Second Harmonic Generation Measurements of Picosecond Band Flattening Processes at Single Crystal Tio2 Electrodes. *The Journal of Physical Chemistry* **1994,** *98* (38), 9387-9390.
14. Hill, D. J.; Teitsworth, T. S.; Ritchie, E. T.; Atkin, J. M.; Cahoon, J. F., Interplay of Surface Recombination and Diode Geometry for the Performance of Axial P–I–N Nanowire Solar Cells. *ACS Nano* **2018,** *12* (10), 10554-10563.
15. Christesen, J. D.; Zhang, X.; Pinion, C. W.; Celano, T. A.; Flynn, C. J.; Cahoon, J. F., Design Principles for Photovoltaic Devices Based on Si Nanowires with Axial or Radial P–N Junctions. *Nano Letters* **2012,** *12* (11), 6024-6029.
16. Gabriel, M. M.; Grumstrup, E. M.; Kirschbrown, J. R.; Pinion, C. W.; Christesen, J. D.; Zigler, D. F.; Cating, E. E. M.; Cahoon, J. F.; Papanikolas, J. M., Imaging Charge Separation and Carrier Recombination in Nanowire P-I-N Junctions Using Ultrafast Microscopy. *Nano Letters* **2014,** *14* (6), 3079-3087.
